# Paralysie périodique hypokaliémique thyrotoxique chez deux femmes noires africaines

**DOI:** 10.11604/pamj.2020.37.207.24900

**Published:** 2020-11-02

**Authors:** Maïmouna Sow, Nafissatou Diagne, Boundia Djiba, Baïdy Sy Kane, Mouhamed Dieng, Awa Cheikh Ndao, Atoumane Faye, Abdoulaye Pouye

**Affiliations:** 1 Département de Médecine Interne, Hôpital universitaire de Dantec, Dakar, Sénégal

**Keywords:** Paralysie, hypokaliémie, hyperthyroïdie, Afrique, Paralysis, hypokalemia, hyperthyroidism, Africa

## Abstract

La paralysie périodique hypokaliémique thyrotoxique est une complication rare de l'hyperthyroïdie. Plus souvent rapportée chez les sujets asiatiques, elle est peu décrite dans la population noire. Son mécanisme peu élucidé, serait lié à une hyper activité de la pompe Na+/K+. Nous présentons deux cas de cette affection survenant chez des sujets noirs africains. La présentation clinique était identique chez les deux malades. Elle était faite d´une paralysie musculaire proximal des membres inférieurs. Cette paralysie était associée à une hypokaliémie sévère et survenait chez des patientes suivies pour maladie de Basedow sans autre pathologie associée. L´évolution a été rapidement favorable sous supplémentation potassique. Le traitement de l´hyperthyroïdie a permis d´éviter les récidives. Ces observations montrent l´importance d´évoquer le diagnostic de paralysie périodique hypokaliémique thyrotoxique malgré la rareté dans la population noire africaine.

## Abstract (en)

Thyrotoxic hypokalemic periodic paralysis is a rare complication of hyperthyroidism. It has been most often reported in Asian subjects while it has been little described in the black population. Its mechanism has been little elucidated, but it would be caused by hyperactivity of the Na+/K+pump. We here report two cases of thyrotoxic hypokalemic periodic paralysis in black African subjects. The clinical manifestation was identical in both patients: proximal muscle paralysis of the lower limbs. Paralysis was associated with severe hypokalemia and occurred in female patients treated for Graves' disease without any other associated disease. Outcome was immediately favorable under potassium supplementation. Treatment of hyperthyroidism prevented recurrences. This study highlights the importance of suspecting the diagnosis of thyrotoxic hypokalemic periodic paralysis despite its rarity in the black African population.

## Introduction

La paralysie périodique hypokaliémique thyrotoxique (PPHT) est une complication rare et potentiellement grave de l´hyperthyroïdie [1]. Elle est plus fréquente dans la population asiatique et rarement décrite chez les sujets noirs [2,3]. Son mécanisme reste peu élucidé mais serait lié à une hypersensibilité de la pompe Na+/K+ ATPase. Elle est caractérisée par une paralysie musculaire et une hypokaliémie dans un contexte d´hyperthyroïdie [1,4]. Des facteurs déclenchants sont souvent retrouvés [2,3]. La PPHT est plus fréquemment décrite au cours de la maladie de Basedow. Toutefois, elle peut survenir au cours des autres causes d´hyperthyroïdie [5]. Nous rapportons deux cas de PPHT dans une population noire africaine.

## Patient et observation

**Première observation:** une jeune patiente sénégalaise de 26 ans était suivie pour maladie de Basedow depuis 3 ans, traitée par le carbimazole (60mg/j) et propanolol (40mg/j). Elle a été admise la nuit aux urgences médicales pour un premier épisode de déficit moteur des membres inférieurs d’installation brutale. Elle rapportait une rupture thérapeutique de 10 jours. L’examen physique montrait un déficit musculaire proximal des membres inférieurs (force musculaire cotée à 1/5), un déficit des muscles extenseurs du cou avec une attitude de nuque tombante. Les réflexes ostéo-tendineux étaient diminués. Il n’y avait pas d’atteinte des muscles respiratoires ou oculaires, ni de troubles sensitifs, ni d’atteinte des nerfs crâniens. L’interrogatoire ne retrouvait aucun facteur déclenchant de l’accès de paralysie. Elle présentait aussi un goitre diffus, régulier, vasculaire et une exophtalmie bilatérale, symétrique et réductible. Il n’existait pas de signe de thyrotoxicose. L’ionogramme sanguin retrouvait une hypokaliémie sévère (K+ <1mmol/l). Le taux de TSHus était effondré <0,0001 UI (0,25 - 5) et le taux de T4 normal. La supplémentation potassique par voie intraveineuse a été instaurée. Le traitement par carbimazole et propanolol a été poursuivi. L’évolution fut marquée par une régression complète du déficit moteur. Le taux de potassium de contrôle était normal à 4,4 mmol/l. Le suivi ultérieur n’a pas révélé de récidive de la symptomatologie.

**Deuxième observation:** une femme sénégalaise de 47 ans nous a été adressée pour prise en charge d’une hyperthyroïdie. Elle avait été hospitalisée en cardiologie pour cardiothyréose avec fibrillation auriculaire et insuffisance cardiaque globale ayant motivé un traitement par diurétiques. Elle a également reçu des perfusions de sérum glucosé 10% pour des épisodes d’hypoglycémie. Dans ses antécédents, elle avait présentait des épisodes de myalgie avec déficit moteur des membres inférieurs spontanément régressifs. A son examen, nous avons noté un déficit musculaire proximal des membres inférieurs (force musculaire à 2/5), sans atteinte de la musculature axiale ni d’atteinte des muscles respiratoires ou oculaires. Les réflexes ostéo-tendineux étaient diminués et il n’y avait pas de troubles sensitifs ni d’atteinte des nerfs crâniens. Elle présentait aussi un syndrome de thyrotoxicose, une exophtalmie bilatérale, asymétrique et réductible et une tachyarythmie auscultatoire. L’ionogramme sanguin montrait une hypokaliémie sévère à 1,9 mmol/l. La calcémie, l’urémie et le taux de créatinine phosphokinase étaient normaux. Le taux de T4 était élevé >100mUI/l (10 - 25) et le taux de TSHus effondré <0,005mUI/l (0,25 - 5). L’échographie cervicale objectivait un goitre hétéro-nodulaire hypervascularisé. Une supplémentation potassique par voie intraveineuse a été faite. Un traitement par antithyroïdiens de synthèse (benzythiouracile 150mg/j) et bétabloquant a été instauré. L’évolution en hospitalisation a été favorable avec amélioration de la force musculaire cotée à 4/5 et correction de l’hypokalièmie (K+ à 4,6).

## Discussion

Nous avons rapporté deux cas de PPHT. La PPHT est une affection rare, décrite notamment chez des jeunes sujets asiatiques [5]. Elle est rare chez les caucasiens [4]. Chez les sujets noirs, les cas sont essentiellement issus d´Amérique du nord [3,6]. Dans la population africaine, quelques observations ont été rapportées dans la littérature [7,8]. Les rares séries rapportées montraient une prédominance masculine de l´affection et un âge de survenue plus bas comparativement aux patients avec hyperthyroïdie, sans PPHT [5,9]. Toutefois, nos observations portent sur deux sujets de genre féminin.

Sa physiopathologie est peu élucidée. Elle serait liée à une hypersensibilité de la pompe NA+/K+ ATPase. Au cours de l´hyperthyroïdie, il y´a une hyper activité de cette pompe. Ceci provoque un transfert inapproprié du potassium vers le milieu intra cellulaire, d´où l´hypokaliémie. Ce transfert est responsable d´un abaissement du potentiel membranaire et de l´hyperexcitabilité musculaire [2]. Les hormones thyroïdiennes inhiberaient le canal potassique Kir 2.6 [1]. Une récente étude montre l´existence de terrain génétique (un haplotype majeur de KCNJ18) de susceptibilité à la PPHT. Ceci expliquerait la plus grande fréquence de cette affection dans la population asiatique [10]. De facteurs déclenchants tels qu´un apport important d´hydrate de carbone, un stress, un exercice physique intense sont fréquemment rencontrés. L´apport d´hydrate de carbone entraine une hyperinsulinémie avec transfert du potassium en intra cellulaire. Le stress provoque une décharge adrénergique avec augmentation de l´activité de la pompe NA+/K+ ATPase [3]. Pour une de nos patientes, aucun facteur déclenchant n´avait été retrouvé. Dans la deuxième observation, un apport d´hydrate de carbone a été noté. Elle avait aussi reçu un traitement par des diurétiques de l´anse ayant pu aggraver l´hypokaliémie.

L´association d´une paralysie, à une thyrotoxicose et une hypokaliémie constitue la triade diagnostique de la PPHT. La présentation clinique est sous la forme d´accès périodique de paralysie flasque atteignant préférentiellement les membres inférieurs. L´installation est souvent brutale et l´intensité variable [2]. La paralysie des muscles respiratoires, potentiellement grave, est rare [2,3,5]. Dans nos observations, aucun cas d´atteinte respiratoire n´a été noté. La présentation clinique était une paralysie musculaire des membres inférieurs d´installation brutale. Le diagnostic de PPHT a été posé lors du premier accès de paralysie dans un de nos cas. Comme retrouvé dans notre étude, l´hypokaliémie est classiquement la seule anomalie métabolique associée à l´hyperthyroïdie [1,2]. La PPHT est le plus souvent associée à la maladie de Basedow [5]. Cette constatation a été également retrouvée dans nos observations.

La prise en charge repose sur une supplémentation potassique associée à un traitement de l´hyperthyroïdie. Dans les formes sévères, l´apport de potassium se fait par voie intraveineuse lente pour prévenir la survenue de troubles du rythme ventriculaire et la paralysie des muscles respiratoires [1]. Le traitement curatif de l´hyperthyroïdie permet de prévenir les récidives. Dans notre travail, un apport de potassium par voie intraveineuse a été proposé à toutes les patientes. Pour la prise en charge de l´hyperthyroïdie, un traitement médical par anti thyroïdiens de synthèse et propanolol a été instauré. Dans plusieurs publications, la même option thérapeutique a été appliquée [4,6]. D´autres équipes ont proposé un traitement chirurgical après l´obtention d´une euthyroïdie [2]. Ces observations soulignent l´importance d´un traitement adéquat de l´hyperthyroïdie, qu´il soit médical ou chirurgical et une bonne éducation thérapeutique pour prévenir cette complication grave. L´évolution rapidement favorable telle que rapportée dans nos observations après supplémentation potassique est la règle [3,4,6].

## Conclusion

Notre travail illustre deux rares cas de PPHT dans une population africaine. La survenue d´une paralysie musculaire flasque d´installation rapide dans un contexte d´hyperthyroïdie a orienté vers une PPHT. La mise en évidence de l´hypokaliémie et l´évolution rapidement favorable après une supplémentation potassique a permis de confirmer le diagnostic. La maladie de basedow était l´étiologie de l´hyperthyroïdie dans toutes nos observations. Sous traitement par antithyroïdiens de synthèse, aucune récidive n´a été notée.
